# Forms of application of silicon in quinoa and benefits involved in the association between productivity with grain biofortification

**DOI:** 10.1038/s41598-022-17181-4

**Published:** 2022-07-26

**Authors:** Luis Felipe Lata-Tenesaca, Renato de Mello Prado, Marisa de Cássia Piccolo, Dalila Lopes da Silva, José Lucas Farias da Silva, Gabriela Eugenia Ajila-Celi

**Affiliations:** 1 Departamento de Fitopatologia, Universidade Federal de Viçosa (UFV), Viçosa, Minas Gerais 36570-090 Brazil; 2 Departamento de Ciências da Produção Agrícola, Universidade Estadual Paulista (Unesp), Jaboticabal, São Paulo 14884-900 Brazil; 3 Centro de Energia Nuclear na Agricultura (CENA), Universidade de São Paulo (USP), Piracicaba, São Paulo 13416-000 Brazil; 4 Departamento de Biologia Aplicada à Agricultura, Universidade Estadual Paulista (Unesp), Jaboticabal, São Paulo 14884-900 Brazil

**Keywords:** Plant development, Plant physiology

## Abstract

Multiple aspects of the physiological and nutritional mechanisms involved with silicon (Si) absorption by quinoa plants remain poorly investigated, as well as the best way of supplying this element to crops. Thus, this study aimed at evaluating whether the application of Si increases its uptake by quinoa plants and consequently the use efficiency of N and P, as well as the levels of phenolic compounds in the leaves, crop productivity and the biofortification of grains. For this purpose, the concentration of 3 mmol L^−1^ of Si was tested, according to the following procedures: foliar application (F), root application in the nutrient solution (R), combined Si application via nutrient solution and foliar spraying (F + R), and no Si application (0). The provision of Si through the leaves and roots promoted the highest uptake of the element by the plant, which resulted in an increased use efficiency of N and P. Consequently, such a higher uptake favored the productivity of grains. The optimal adoption of the application of Si through leaves and roots promoted the highest Si concentration and ascorbic acid content in quinoa grains.

## Introduction

The cultivation of quinoa plants has been gaining prominence over the last decades, due to its potential benefits to human health, as it contains all the essential amino acids considered for human nutrition, as well as high levels of minerals and vitamins^1,2^. These traits can also be related to the prevention of many diseases^3,4^. However, it is possible to expand even more the benefits of quinoa through biofortification with silicon (Si), given the beneficial properties that this element can promote in plants.

Silicon is considered an essential element for humans, playing a fundamental role in bone metabolism and in the functions of the nervous and immune systems^5,6^. Therefore, the provision of Si to crops such as quinoa could be a feasible way of obtaining biofortified grains, thus improving its nutritional quality. Si is also known to increase the content of ascorbic acid in plants, which is an important antioxidant^7^ and a source of vitamin C^8^. This is an important feature of quinoa grains, because its levels of ascorbic acid are relatively low, ranging from 5.0 to 10 mg 100 g^−1^^9,10^.

The process of grains biofortification is an important practice to improve the nutritional quality of foods, even though it is a relatively recent field. Thus, this process must be thoroughly investigated in terms of increasing both nutritional quality of crops and their productivity. In this sense, the production chain can be strengthened as a whole, especially considering small farmers, seen that their remuneration is quite restrict and measured by their productivity, instead of considering also the quality of grains. Therefore, innovations are made necessary regarding the process of biofortification, in order to associate it with productivity. For this purpose, the ways Si can be applied to crops should be better evaluated, in the attempt of increasing its uptake by plants.

Si can be applied via nutrient solution or fertigation, aiming to promote its uptake by roots, as well as through foliar spraying, in order to increase its uptake by leaves and grains^11^. Foliar spraying can be performed during the vegetative and reproductive period of the plants; and a study that investigated foliar application of Si in soybean plants reported higher concentration in its grains, in addition to an increased productivity^12^. However, when provided via the root system in a nutrient solution, the concentration of Si was found to be three times higher in green bean pods^13^. In vegetables, this element is considered biologically accessible in an in vitro digestion process, therefore it is consequently an important source of the element for humans^14^.

Several studies demonstrated the effects of Si in relation to increased crops development and productivity^15,16^. These beneficial effects are made possible due to higher nutrient uptakes (e.g. N and P), which favors biomass production in multiple crops, such as sugarcane^17^ and wheat^18^. Another benefit of Si is the production of antioxidant compounds (e.g. phenols), as reported in barley plants^19^, which may favor the plant's metabolism and consequently crop productivity as a whole, even though this effect might depend on the capacity of the plant to absorb Si.

The ability of quinoa plants to absorb Si was very little investigated hitherto; however, a previous study indicated that this species has the capacity to absorb this element in sufficient amounts to display increased productivity^20^. Nevertheless, a knowledge gap exists in relation to the effects of Si application in quinoa plants, thus the following hypothesis were considered in this study: (i) quinoa plants absorbs Si and such absorption can be enhanced when applying this element via roots in association with foliar fertilization, in comparison to its isolated application; and (ii) if so, the higher uptake of Si by quinoa plants will increase the levels of antioxidant compounds, as well as the use efficiency of N and P, favoring its productivity and grain quality. In case these hypotheses are accepted, it might be possible to expand the knowledge on the mechanisms behind Si absorption in an important commercial plant species, that is quinoa.

The aim of this study was to evaluate whether Si application increases its uptake by quinoa plants and consequently the use efficiency of N and P, as well as the levels of phenolic compounds in the leaves of quinoa plants, crop productivity and the biofortification of grains.

## Results and discussion

### Silicon absorption and accumulation in quinoa plants

**Figure 1:**
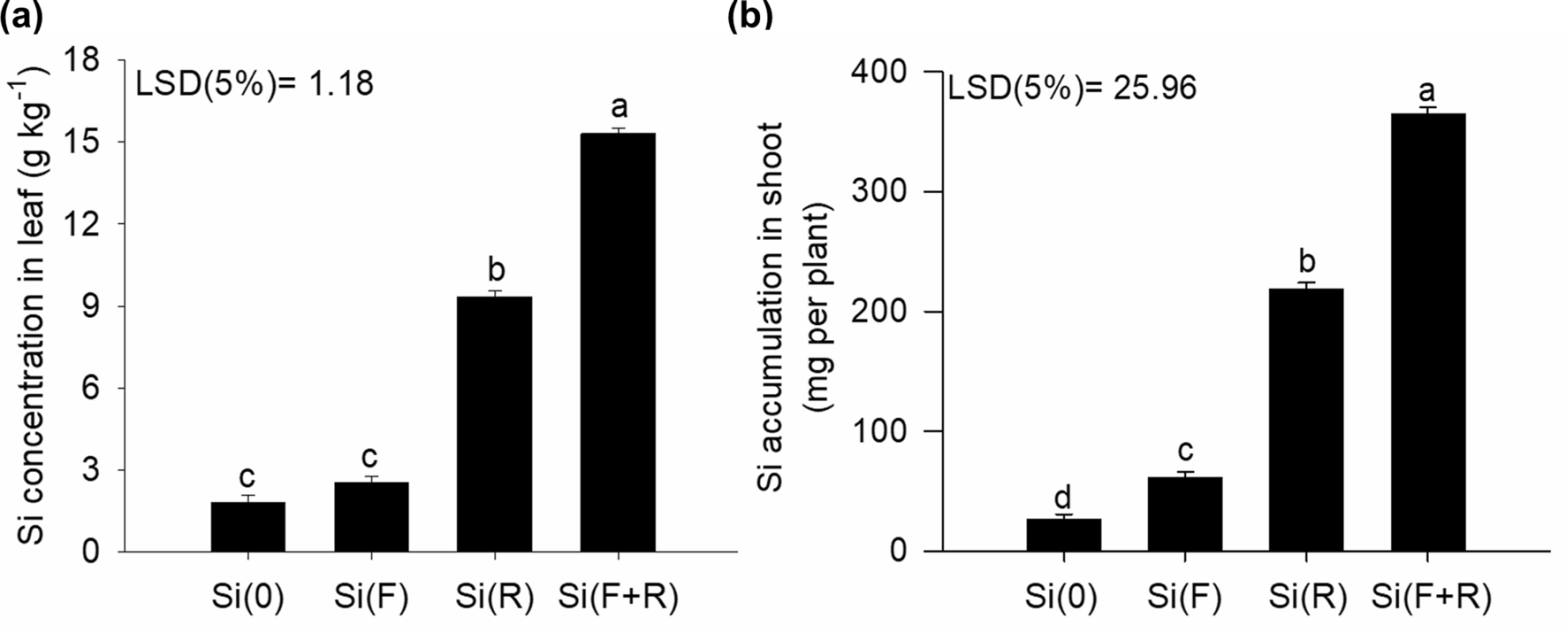
Concentration of Si in the leaves (**a**) and Si accumulation in shoots (**b**) of quinoa plants, according to different forms of Si application; no Si [Si(0)]; foliar Si application [Si(F)]; root Si application [Si(R)]; combination between root and foliar application [Si(F + R)]. Letters indicate significant differences between the forms of Si application (p < 0.05); least significant difference [LSD]. The vertical bars represent the standard error of the mean.

The absorption of Si has been widely investigated when this element is applied via the root system^21^, and plants can be classified either as non-accumulators (< 5 g kg^−1^), intermediate (5 to 10 g kg^−1^), or accumulators of Si (> 10 g kg^−1^)^22^, depending on the concentration of Si found in the leaves. The concentration of Si in leaves as a function of root application of Si with the nutrient solution [Si(R)] was found to be 9.3 g kg^−1^ (Fig. 1a). This information was demonstrated in this study because an increment in the accumulation of Si was observed in the shoots (Fig. 1b), especially in the treatment combining application of Si via nutrient solution and foliar spraying [Si(F + R)] at the concentration of 3 mmol L^−1^, which stood out in relation to the other treatments, although the foliar application was statistically different from the control. Therefore, it is evident from this information that quinoa accumulates intermediate levels of Si, which agrees with previous studies^20,23^. This result proves part of the first hypothesis raised in this study, indicating that the foliar application of Si, in association with the roots can enable a higher Si absorption in this plant species. This effect indicates the importance of a continuous supply of Si throughout the entire developmental cycle via nutrient solution, in order to complement it with foliar applications, seen that foliar applications alone do not promote a higher absorption of this beneficial element.

### Silicon increases the absorption of N, P and antioxidant compounds

**Figure 2:**
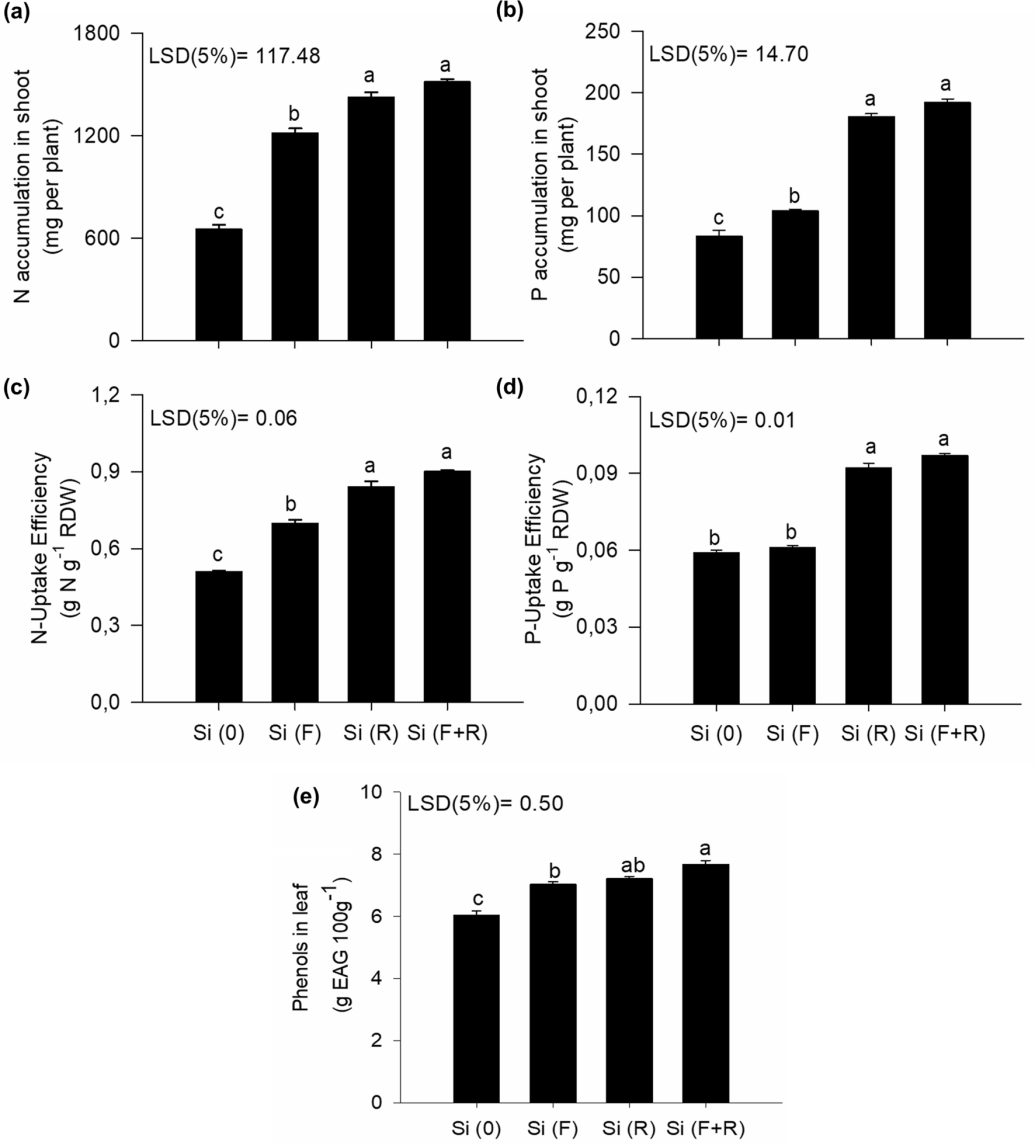
Accumulation of N (**a**), and P in the shoots (**b**), uptake efficiency of N (**c**) and P (**d**) and phenolic compounds in the leaves (**e**) of quinoa plants, according to different forms of Si application; no Si [Si(0)]; foliar Si application [Si(F)]; root Si application [Si(R)]; combination between root and foliar application [Si(F + R)]. Letters indicate significant differences between the forms of Si application (p < 0.05); least significant difference [LSD]. The vertical bars represent the standard error of the mean.

Increased uptake efficiencies and accumulations of N and P were observed when Si was provided, highlighting that both treatments Si(F + R) and Si(R) supplied the element in a similar way (Fig. 2a–d). In addition, the influence of the forms of Si application on the production of phenolic compounds in leaves was evident (Fig. 2e). Hence, the effect of Si in increasing the uptake of N, P and phenols in leaves was visible. The benefit of Si in increasing N uptake was reported in sugarcane^17^, and of P in wheat crops^18^, which can be explained by the fact that Si acts in the regulation of photosynthesis and transpiration, and it increases the expression of genes related to nutrient transporters in cellular membranes. It was also reported that Si supply in quinoa plants attenuated the effects of N and P deficiency by preserving the photosynthetic apparatus and decreasing the electrolyte leakage^23^. In this context, future studies at the molecular level should be conducted to further understand the potential role of Si in the regulation of important physiological processes in quinoa, such as the processes of N and P uptake, nutrient uptake by the plant and impacts on grains.

We evidenced that the combined application of Si(F + R) was associated with high content of phenolic compounds in leaves, in comparison to the treatments foliar application [Si(F)] and no Si application [Si(0)]. This information is relevant, since it was indicated that quinoa leaves can also be consumed as cooked vegetables and as a salad, increasing its interest as a functional food^24^. An increased production of phenolic compounds due to Si application has been reported in cucumber^25^ and barley crops^19^. Those authors highlighted the action of Si in modulating the metabolism of phenols by stimulating the formation of Si polyphenol complexes and via the regulation of enzymes involved to phenylpropanoid pathway^26^.

### Silicon improves grain productivity and quality

**Figure 3:**
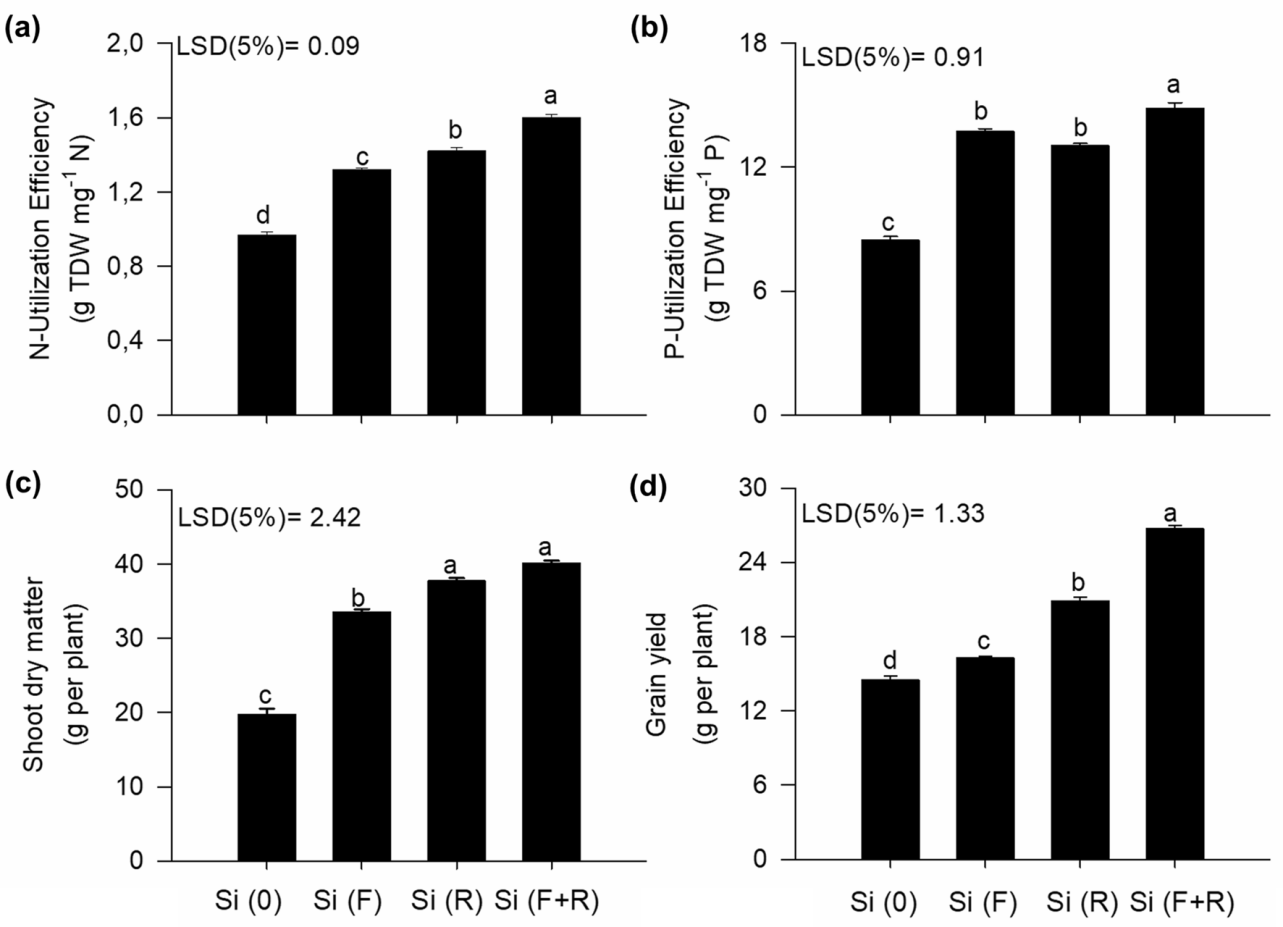
Use efficiency of N (**a**) and P (**b**), shoot dry mass (**c**) and grain productivity (**d**) in quinoa grains, according to different forms of Si application; no Si [Si(0)]; foliar Si application [Si(F)]; root Si application [Si(R)]; combination between root and foliar application [Si(F + R)]. Letters indicate significant differences between the forms of Si application (p < 0.05); least significant difference [LSD]. The vertical bars represent the standard error of the mean.

In this study, both treatments Si(F + R) and Si(R) resulted in a significantly higher production of dry mass in the shoots of quinoa plants, while grain productivity was significantly improved by the combined application of Si(F + R), in comparison to the treatments Si(F) and Si(R) (Fig. 3c,d). The application of Si(F + R) can be considered promising, given the response of the plant evidenced by the increase in grain productivity by 84% in comparison to the control treatment. This effect occurred due to the best use of Si by the plants that received foliar and root supply (F + R) of this element, which resulted in a greater uptake of the element by plants, thus resulting in an increased use efficiency of both N and P (Fig. 3a,b). These nutrients displayed more beneficial effects in the plants metabolism and stimulated its biological functions, which in turn led to a higher grain production. These results are supported by the positive correlation among Si accumulation in the shoots, N and P use efficiency, and quinoa grain productivity (Supplementary Fig. S1). Therefore, this finding corroborates the second hypothesis raised in the study, indicating that the greater Si uptake by quinoa plants might favor grain production.

**Figure 4:**
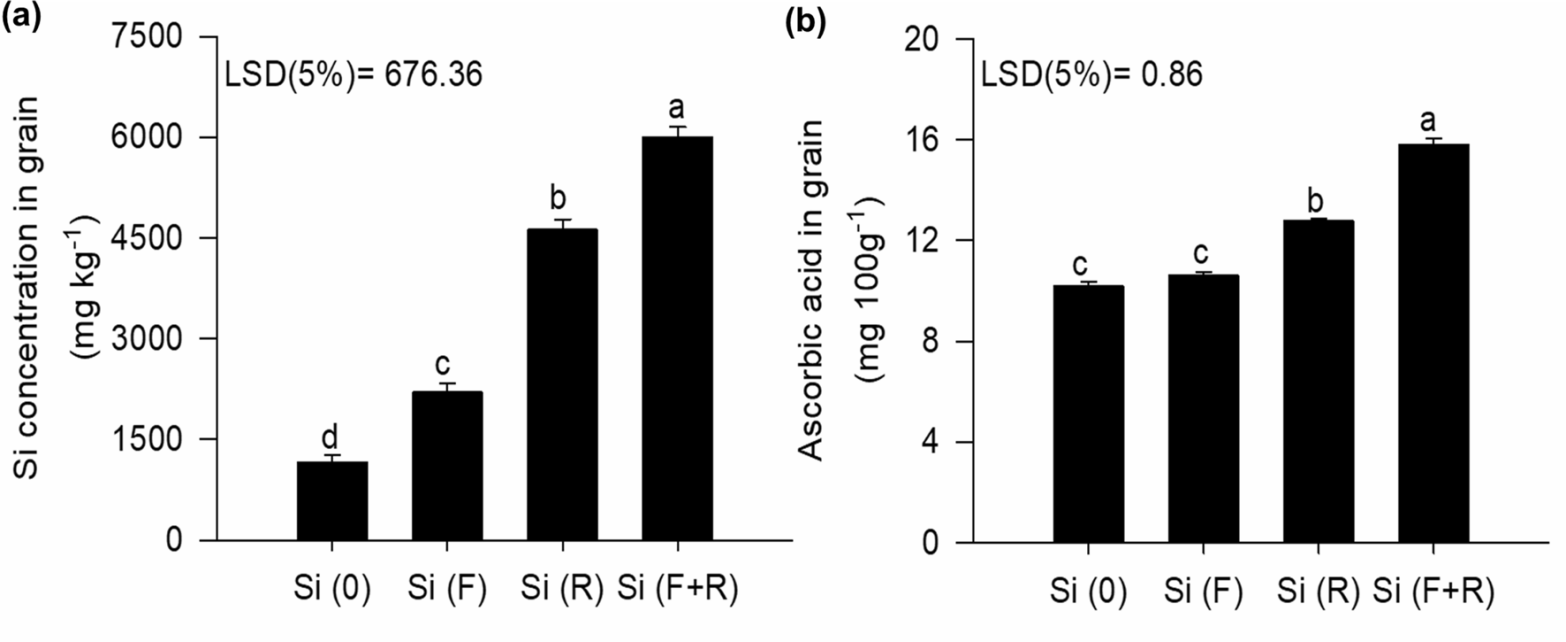
Si (**a**) and ascorbic acid (**b**) concentrations in quinoa grains, according to different forms of Si application; no Si [Si(0)]; foliar Si application [Si(F)]; root Si application [Si(R)]; combination between root and foliar application [Si(F + R)]. Letters indicate significant differences between the forms of Si application (p < 0.05); least significant difference [LSD]. The vertical bars represent the standard error of the mean.

In addition to the benefits of Si in the productivity of quinoa, the results presented herein demonstrated that both forms of application of Si (R and F + R) led to respective increases of 110 and 170% of the Si concentration in the grains, in comparison to plants that received singular foliar applications of the element (Fig. 4a). Therefore, it was evidenced that adopting an optimized form of Si application (F + R) enables grain biofortification with Si. It should be noted that the average consumption of quinoa per person to meet part of the daily nutritional recommendation of Si is 40 g^27^, and the minimum recommended Si intake for adults is 50 mg per day^28^. Considering the positive results of Si application in quinoa plants through its leaves, roots, and the combination of both forms in accumulating this element in grains, the obtained accumulation rates could contribute to a daily intake of 76, 155, and 205 mg of Si, while grains derived from control plants would give an intake of 39 mg Si per day, thus not meeting the minimum daily requirement of Si for humans.

Quinoa grains have low contents of ascorbic acid, but when an optimized Si application was performed Si(F + R), the ascorbic acid content reached a maximum value of 15.8 mg 100 g^−1^ (Fig. 4b), which is a much higher concentration in comparison to other studies, such as 10 mg 100 g^−1^^10^ and 7 mg 100 g^−1^^29^. However, this effect has been reported in rice^30^, chard and kale^31^, possibly due to the fact that Si stimulates the production of non-enzymatic antioxidants, such as ascorbate. This finding is unprecedented in studies on Si biofortification, indicating a supplementary effect of this element in increasing the ascorbic acid content, thus solving this deficiency of quinoa, which is a poor dietary source of this vitamin.

**Figure 5:**
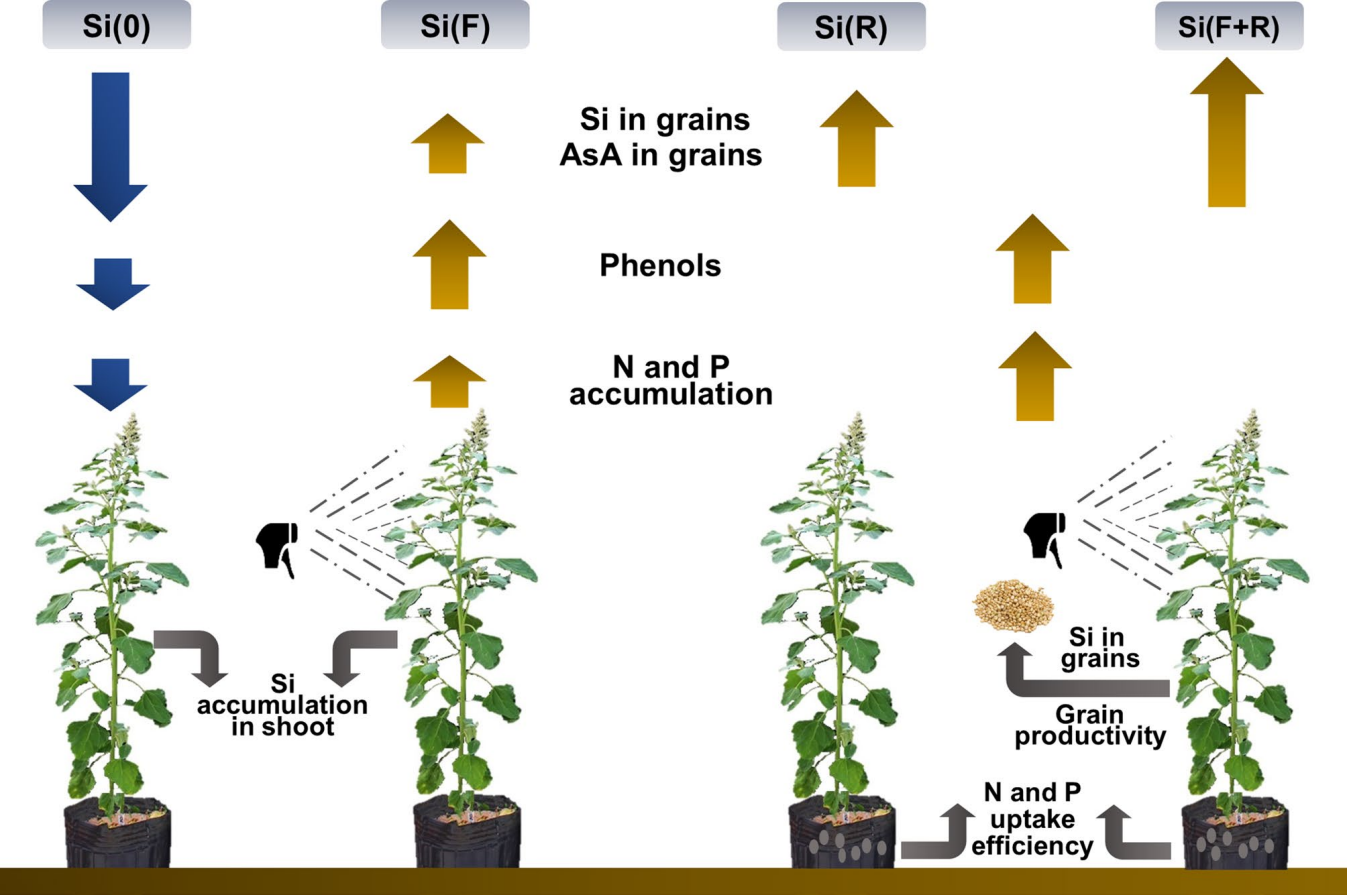
Graphical abstract of the forms of application of Si in quinoa and main results in productivity and grain quality.

The findings of this study elucidated that quinoa can be considered an intermediate plant species regarding Si uptake. Therefore, this research shows that optimizing the Si application, i.e., via roots in association with foliar fertilization is a new strategy to improve quinoa productivity and strengthen the quality of these grains for human nutrition (Fig. 5, Supplementary Fig. S1). However, future trials would be needed to tailor this strategy under field conditions in combination with others Si sources and concentrations.

## Methods

### Experimental conditions and plant growth

**Figure 6:**
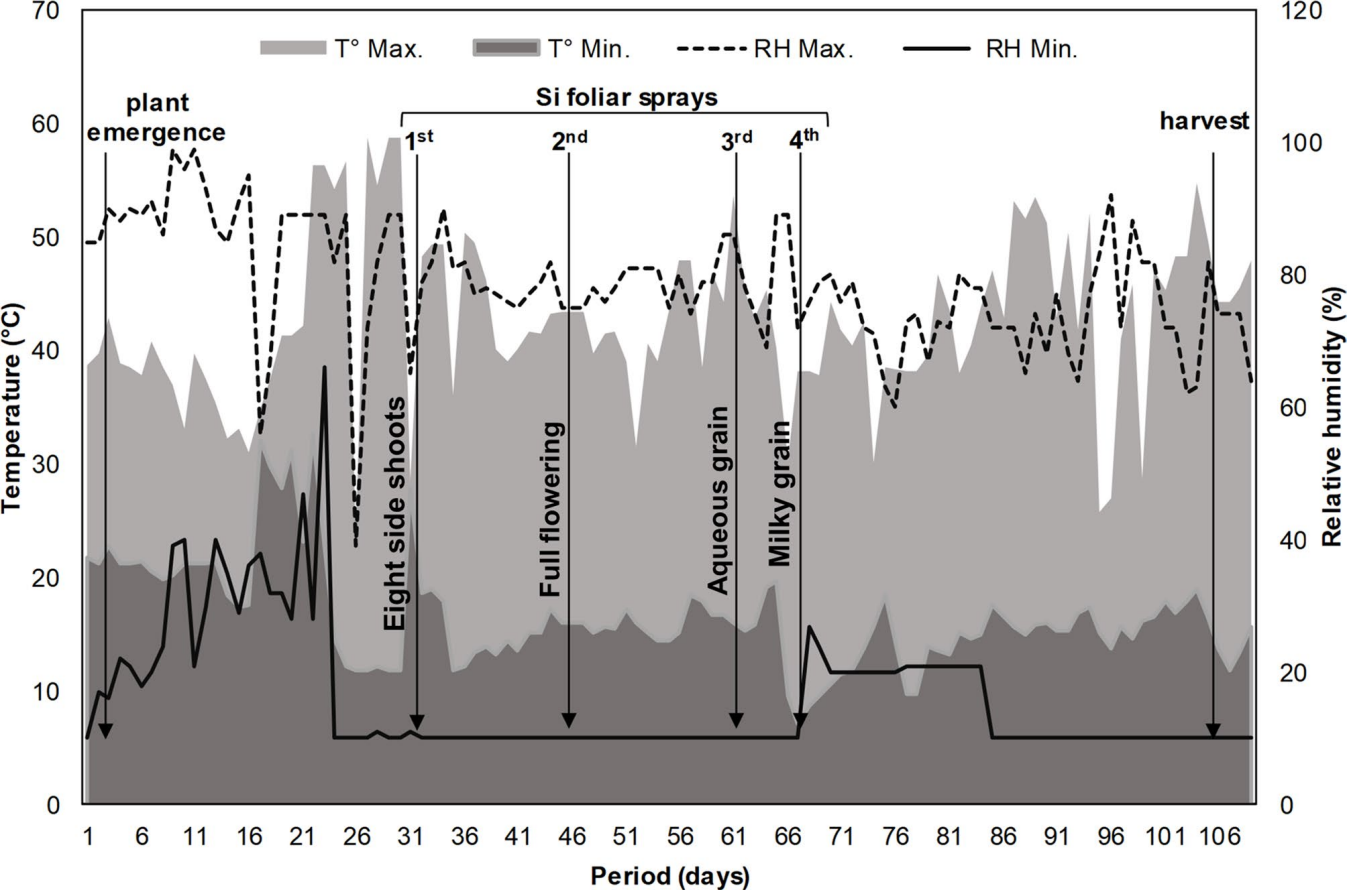
Experimental conditions inside the greenhouse used in this study. Maximum temperature (T° Max.), minimum temperature (T° Min.), maximum relative humidity (RH Max.), and minimum relative humidity (RH Min.).

The experiment was conducted between May and August 2019 in a hydroponic cultivation system, inside a greenhouse and under natural photoperiod at São Paulo State University, city of Jaboticabal, Brazil. Seeds of quinoa cv. BRS Piabiru^32^, were obtained from the Brazilian Agricultural Research Corporation of the Ministry of Agriculture, Livestock and Food Supply, Brazil. This research was not conducted with endangered species and was carried out in accordance with the Declaration of the IUCN Policy on Research Involving Endangered Species. The temperature registered inside the greenhouse was 29.7 ± 4.15 °C throughout the experiment, and the relative humidity was 47 ± 32% (Fig. 6).

Stabilized sodium and silicate with sorbitol were used as the source of Si for the foliar application and the nutrient solution in this study [Si = 107.9 g L^−1^; K_2_O = 16.44 g L^−1^; Na_2_O = 60.7 g L^−1^, pH 11.8]. Sorbitol plays an indispensable role as Si stabilizer in aqueous solutions maintaining the Si monomeric forms by forming organic complexes process and decreases the deliquescence point of the droplet on the leaf surface^33^. The concentration of K in both the nutrient solution and the foliar application was balanced by using KCl among treatments. The concentration of 3 mmol L^−1^ of Si was tested, according to the following procedures: foliar application (F); root application with the nutrient solution (R); combined application of Si via nutrient solution and foliar spraying (F + R); and no Si application (0). These types of application were arranged in a randomized blocks design, with five replicates per treatment for a total of 20 plants. The concentration of 3 mmol L^−1^ was chosen because it would be the maximum concentration without the risk of polymerization of the element^34^.

Ten quinoa seeds were arranged in 6.0 dm^3^ polyethylene pots filled with washed inert sand. After germination, the seedlings were cut, so that a single plant was kept per pot until the end of the experiment. The sand was washed with running tap water and then with deionized and distilled water. After seedling emergence, the nutrient solution proposed by Hoagland and Arnon^35^ was used with some modifications: 7.5 mM NH_4_NO_3_, 0.5 mM KH_2_PO_4_, 3.0 mM KNO_3_, 5.0 mM Ca(NO_3_)_2_, 1.0 mM MgSO_4_, 1.0 mM (NH_4_)_2_SO_4_, 23 µM H_3_BO_3_, 0.15 µM CuCl_2_; 45 µM Fe-EDDHA, 6.3 µM MnCl_2_ 4 H_2_O, 0.05 µM H_2_MoO_4_H_2_O, and 0.65 µM ZnCl_2_. The ionic strength of the solution started with 10% during the first 10 days after emergence, then was increased to 25% throughout the initial vegetative phase (up to eight permanent leaves), and then increased once again to 50% until the beginning of the flowering period. Subsequently, it was increased to 80% until grains began to appear, and then once again increased to 100% until the end of the experiment. The pH value of the nutrient solution was daily adjusted to 5.5 ± 0.2, by using either a HCl or NaOH solution (1 N).

Four foliar sprayings were performed with Si in the following stages^36^: eight side shoots visible, full flowering, and in the stage during which aqueous and milky grains were observed (see Fig. 6). Foliar applications were performed in the morning (from 6h00 to 7h00), when the relative air humidity ranged between 70 and 90% and the temperature was found between 15 and 18 °C. These conditions are considered adequate for the adoption of foliar spraying^37^. The pH value of the solution used in the foliar application was kept at 7.5 ± 0.5, by means of a HCl solution (1 N), which might induce an increase in monomeric species in the solution^38^. Si does not cause toxicity in plants^39^; therefore, no toxicity symptoms were evident on the leaves with foliar application of Si. The Si applied via the root system was supplied with the nutrient solution throughout the experimental period. The phenological cycle of quinoa lasted for 103 days after emergence, when the plants were harvested for further analysis.

### Plant analysis

Approximately one week before the plants were collected, leaf disks were collected from the most developed leaf of each plant in its upper third. The leaf content of total phenolic compounds was determined followed the methodology proposed by Singleton and Rossi^40^. Briefly, a sample of 100 mg of quinoa fresh matter was placed inside a Falcon tube (15 mL) containing methanol (95%), and the test tube was put in a water bath at 25 °C for 3 h. Subsequently, 1 mL of the diluted sample was reacted with 0.5 mL of Folin-Ciocalteu reagent for 5 min, and then 1.5 mL of sodium carbonate solution (Na_2_CO_3_) was added to the mixture. Absorbance readings were taken at 765 nm after incubation at room air temperature for 2 h.

When the plants reached physiological maturity (ripe grain^36^), they were segmented into roots, stems, leaves and grains, and dried in a forced ventilation oven at a constant temperature of 65 °C until constant weight, and then the dry mass was measured. The concentration of ascorbic acid in the grains was determined by titration^41^. The N content was obtained by means of the dry combustion method (1000 °C), using an elemental analyzer (LECO TruSpec CHNS) that was calibrated with the LECO 502-278 wheat standard (C = 45.00% and N = 2.68%). The P content was determined using the colorimetric antimony method of molybdenum^42^. For the determination of the Si content, the procedure of wet digestion was performed, by adding hydrogen peroxide (H_2_O_2_) and sodium hydroxide (NaOH), with the reaction being induced in an autoclave at 123 °C. The reading of the Si content was performed by the colorimetry method with hydrochloric acid, oxalic acid, and ammonium molybdate^43^.

The accumulated contents of N, P and Si in the shoots were determined by means of the results obtained in dry mass basis, being expressed in mg per plant. The nutrients uptake efficiency was calculated as the total nutrient accumulation divided by the weight of dry roots (g of each element g^−1^ RDW)^44^. Finally, the nutrient use efficiency was calculated as the total plant dry weight (TDW), divided by both N and P content (g TDW mg^−1^ of each element)^45^, while the result of grain productivity was expressed in g per plant, adjusting the moisture content to 15%^32^.

### Statistical analysis

All data were analyzed for normality using the K-S test and homogeneity by means of the Bartlett test. Subsequently, the data obtained from each treatment was submitted to a variance analysis applying the F-test, and means were compared by least significant differences with a 5% significance level (p < 0.05). Statistical analyses were performed with the aid of the software SAS, Version 9.1 (SAS Institute, Cary, NC, USA).

### Statement of handling of plants

The authors confirm that the handling of the plants is accordance with the Declaration of IUCN Policy on Research Involving Endangered Species and the Convention on Trade in Endangered Species of Wild Fauna and Flora.

## Supplementary Information

Supplementary Figure S1.
