# Supplementary material for: Forms of application of silicon in quinoa and benefits involved in the association between productivity with grain biofortification
Source: Sci Rep. 2022 Jul 26;12:12732. doi: 10.1038/s41598-022-17181-4 (PMC9325773; doi:10.1038/s41598-022-17181-4)
Supplement: Supplementary file 1 — Supplementary Figure S1. [file 41598_2022_17181_MOESM1_ESM.docx]

**Forms of application of silicon in quinoa and benefits involved in the association between productivity with grain biofortification**

Luis Felipe Lata-Tenesaca^1*^, Renato de Mello Prado^2^, Marisa de Cássia Piccolo^3^, Dalila Lopes da Silva^3^, José Lucas Farias da Silva^2^ & Gabriela Eugenia Ajila-Celi^4^

^1^ Departamento de Fitopatologia, Universidade Federal de Viçosa (UFV), Viçosa, Minas Gerais, 36570-090, Brazil.

^2^ Departamento de Ciências da Produção Agrícola, Universidade Estadual Paulista (Unesp), Jaboticabal, São Paulo, 14884-900, Brazil.

^3^ Centro de Energia Nuclear na Agricultura (CENA), Universidade de São Paulo (USP), Piracicaba, São Paulo, 13416-000, Brazil.

^4^ Departamento de Biologia Aplicada à Agricultura, Universidade Estadual Paulista (Unesp), Jaboticabal, São Paulo, 14884-900, Brazil.

*Correspondence and requests for materials should be addressed to L.F. Lata-Tenesaca (email: [lfelipelata@gmail.com](mailto:lfelipelata@gmail.com)).

**Supplementary Figure**


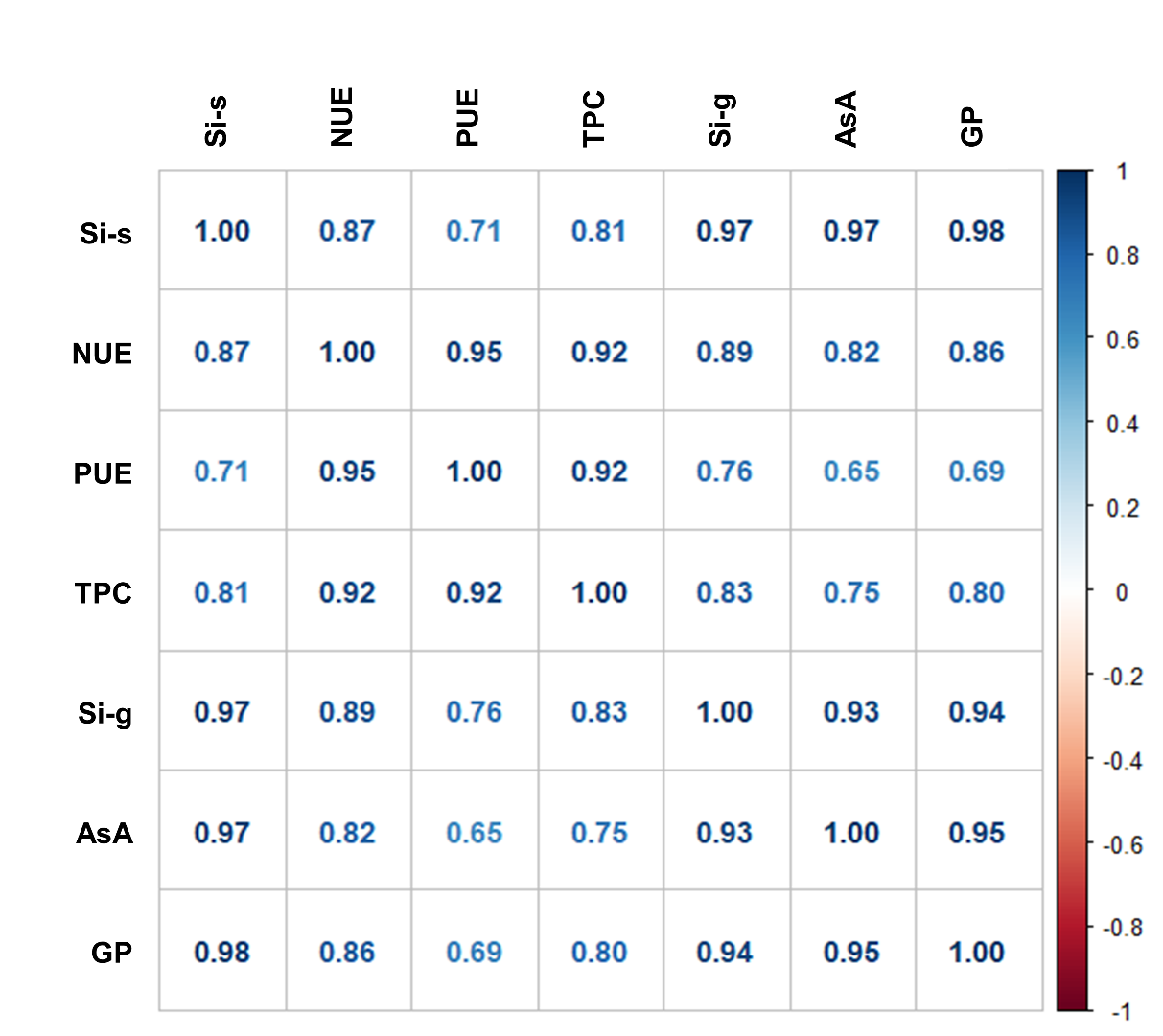


**Figure S1.** Corrplot representing the correlation among Si accumulation, use efficiency of N and P, productivity, and quinoa grain quality. Pearson’s correlation coefficients (p≤0.05) for a sample size n=20, are shown according to positive correlations in blue. The abbreviations are as follows: Si accumulation in shoot (Si-s), N use efficiency (NUE), P use efficiency (PUE), total phenolic compounds (TPC), Si concentration in grain (Si-g), ascorbic acid content in grain (AsA), and grain productivity (GP).
